# Promoting cardioprotection with fenugreek: Insights from CoCl_2_-induced hypoxia in neonatal rat cardiomyocytes

**DOI:** 10.22038/IJBMS.2023.71521.15547

**Published:** 2023

**Authors:** Noorul Izzati Hanafi, Maizan Mohamed, Kuttulebbai Naina Mohamed Salam Sirajudeen, Noor Hafizoh Saidan, Gan Siew Hua, Khomaizon Abdul Kadir Pahirulzaman, Pasupuleti Visweswara Rao

**Affiliations:** 1 Faculty of Agro-Based Industry, Universiti Malaysia Kelantan, Jeli, Kelantan, Malaysia; 2 Faculty of Veterinary Medicine, Universiti Malaysia Kelantan, Pengkalan Chepa, Kelantan, Malaysia; 3 Department of Basic Medical Sciences, Kulliyyah of Medicine, International Islamic University Malaysia (IIUM), Bandar Indera Mahkota, Kuantan, Pahang, Malaysia; 4 School of Pharmacy, Monash University Malaysia, Jalan Lagoon Selatan, 47500 Bandar Sunway, Selangor, Malaysia; 5 Department of Biomedical Sciences and Therapeutics, Faculty of Medicine and Health Sciences, Universiti Malaysia Sabah, Kota Kinabalu, 88400, Sabah, Malaysia; 6 Department of Biochemistry, Faculty of Medicine and Health Sciences, Abdurrab University, Pekanbaru, Riau, Indonesia; 7 Centre for International Relations and Research Collaborations, Reva University, Rukmini Knowledge Park, Kattigenahalli, Yelahanka, Bangalore, 560064, Karnataka, India

**Keywords:** Cardiomyocytes, Hypoxia, Ischemia, Therapeutics, Trigonella foenum-graecum

## Abstract

**Objective(s):**

This study aimed to investigate the protective effects of fenugreek on CoCl_2_-induced hypoxia in neonatal rat cardiomyocytes.

**Materials and Methods:**

Primary cardiomyocytes were isolated from Sprague Dawley rats aged 0–2 days and incubated with various concentrations of fenugreek (10-320 µg/ml) and CoCl_2_-induced hypoxia for different durations (24, 48, and 72 hr). Cell viability, calcium signaling, beating rate, and gene expression were evaluated.

**Results:**

Fenugreek treatments did not cause any toxicity in cardiomyocytes. At a concentration of 160 µg/ml for 24 hr, fenugreek protected the heart against CoCl_2_-induced hypoxia, as evidenced by reduced expression of *caspases* (-*3*, *-6*, *-8*, and *-9*) and other functional genes markers, such as *HIF-1α*, *Bcl-2*, *IP3R*, *ERK5*, and *GLP-1r*. Calcium signaling and beating rate were also improved in fenugreek-treated cardiomyocytes. In contrast, CoCl_2_ treatment resulted in up-regulation of the hypoxia gene *HIF-1α* and apoptotic caspases gene (*-3*, *-9*,* -8*, *-12*), and down-regulation of *Bcl-2* activity.

**Conclusion:**

Fenugreek treatment at a concentration of 160 µg/ml was not toxic to neonatal rat cardiomyocytes and protected against CoCl_2_-induced hypoxia. Furthermore, fenugreek improved calcium signaling and beating rate and altered gene expression. Fenugreek may be a potential therapeutic agent for promoting cardioprotection against hypoxia-induced injuries.

## Introduction

Functional foods are defined as food components that provide health benefits beyond basic nutrition (1). Many Asian countries use flavorful herbs and spices in their cuisine, which not only enhance the taste but also offer numerous health benefits as functional foods. As the population continues to grow, the importance of consuming healthy functional foods becomes increasingly important to promote good health. Overconsumption of unhealthy foods is linked to numerous diseases and high mortality rates. Consumption of animal meat that is rich in fats is a considerable contributor to the development of several illnesses, such as cardiovascular diseases, which can result in heart-related ailments. According to the World Health Organization (WHO), in 2016, approximately 2 billion adults were overweight, and 650 million were diagnosed with obesity (2).

Individuals who are overweight are more susceptible to cardiometabolic disorders due to poor dietary habits and lack of physical activity. Obesity is a significant independent risk factor that contributes to the development of cardiovascular disease, hypertension, type-2 diabetes, and musculoskeletal disorders (3). An excess amount of fat in the bloodstream interferes with insulin sensitivity, which leads to elevated blood glucose levels. In order to maintain good health, people spend a considerable amount of money on various products such as healthy foods, supplements, and medicines. Medicinal herbs and spices are widely recommended, particularly in developing countries, due to their potential polyphenols or flavonoids with antioxidant properties that can improve general health. This is largely due to the belief that medicinal herbs and spices have fewer side effects than synthetic drugs.

Fenugreek (*Trigonella foenum-graecum*) is one of the oldest medicinal plants that play an important role in promoting a healthy body. This annual herb, which has white flowers and aromatic seeds, is widely used in food preparation. In traditional Chinese medicine, fenugreek was used to treat kidney and liver diseases (4), while in India, it was used in Ayurveda to prevent hair loss and dandruff by moisturizing the hair (5). Fenugreek has antidiabetic properties and can improve blood sugar, kidney, and liver function even at higher doses or longer duration of treatment (6). The plant’s antioxidant properties suggest it may be a potential cardioprotective agent against myocardial infarction by suppressing oxidative responses (7). Additionally, fenugreek has therapeutic properties in metabolism and animal physiology, including as a growth promoter (8, 9). It can lower plasma cholesterol levels (10), improve food intake, stimulate immune functions (11), lower kidney-to-body weight ratio (12), blood sugar (13), and blood lipid levels (14), and improve hemorheological parameters (15). Although systemic studies on the effect of fenugreek on the heart are limited, an *in vitro* study aimed to investigate its mechanistic action as a therapeutic cardioprotective agent that can be taken as a daily dietary supplement for ischemic heart injury.

## Materials and Methods

***Animal and diets***

All protocols in this study were approved by the Institutional Animal Care & Use Committee (IACUC) Universiti Malaysia Kelantan, Malaysia (UMK/FIAT/ACUE/PG1/2018). Female and male Sprague Dawley rats aged 3–6 months were purchased from the Animals Research and Service Centre, Universiti Sains Malaysia, Malaysia. Rats were acclimatized for a minimum of a week before the beginning of the study. The rats were maintained with *ad libitum* food and water on a light dimmer (40 lux, 1 m above floor) at room temperature (26–30 °C) and humidity (40–70%). Provision of 15–20 air changes per hour was provided to maintain room air quality. The cage has wired cover for drinking bottles and food (pellet). Pinewood chips were used as nesting materials. Cleaning the cages to remove excessive amounts of dirt, debris, and disinfection of microorganisms was done every 3 days. Rat cages were placed inside a room to avoid loud sounds or noise. The rats were caged inside 8 cm height boxes that allow rats to stand on their hind legs and stretch up fully. 

For breeding, the mature male and female were placed together. The positive result of vaginal smear cytology identification on Day 5 of mating when the oestrus cycle shows a high pregnancy percentage with the combination of dioestrus (contains leucocytes with a small number of epithelial and cornified cells) and metoestrus (more leucocytes were observed and no non-nucleated epithelial cells). After mating, female rats were separated and housed in individual cages with environmental enrichment to facilitate the natural progression of nesting and littering. On day 21, the female rat delivered approximately 6–12 pups per litter. The 0–2 day-old pups were subjected to further experiments. 

***Neonatal rat cardiomyocytes***

The cardiomyocytes were freshly prepared for each designated experiment using the method described by Sheikh, 2010 (16). The neonates (0–2 days old) of Sprague-Dawley rats were culled by cervical dislocation and decapitation. This technique is used to reduce the pain and preserves the internal organ. The hearts were removed, excised, and immersed in 20 ml artificial dissociation solution (ADS) buffer (116 mM NaCl, 20 mM HEPES, 1 mM NaH_2_PO_4_, 5.5 mM glucose, 5.4 mM KCl, 0.8 mM MgSO_4_, pH 7.35) to remove blood. Ventricles were separated from other tissue using scissors, minced finely in fresh 10 ml ADS buffer, and washed three times. In the final wash, the minced tissue suspension was incubated at 37 °C for 5 min at 230 rpm. Subsequently, cardiomyocytes were detached from the extracellular matrix by repeated digestion and incubation steps (Table 1) in collagenase buffer (1x ADS buffer, 0.2 mg/ml pancreatin, 207 U/ml collagenase II). Cardiomyocytes were pooled from each digestion step in a 50 ml falcon tube and incubated at 95% O_2_ with 5% CO_2_. All pooled cardiomyocytes were centrifuged at 1500 rpm for 3 min. Cells were kept in plating media (DMEM supplemented with 17% Medium 199 (v/v), 10% horse serum (v/v), 5% fetal calf serum (v/v), 200 µg/ml streptomycin, 200 U/ml penicillin). The cardiomyocyte confluence at 70% was required for each experiment. Thus, cells were incubated at 37 °C with 5% CO_2_ for 12 hr. 

***Treatment of fenugreek on cardiomyocytes***

Food-grade fenugreek absolute natural (Sigma Aldrich, USA) was prepared as a stock solution at 1 mg/ml in 0.1 % DMSO and filtered using a 0.2 µm syringe filter. The stock solution was stored at 4 °C. Working solutions were prepared by diluting the fenugreek stock solution in a culture medium at various concentrations ranging from 0 to 320 µg/ml. The optimum effect of fenugreek was evaluated before (pre-fenugreek) or after (post-fenugreek) induction of hypoxia on cardiomyocytes. To induce a hypoxic condition, the cells were treated chemically using cobalt chloride (CoCl_2_). In pre-fenugreek treatment, the cardiomyocytes were incubated for 12 hr with fenugreek followed by treatment with CoCl_2_ and fenugreek for 24 hr. Whereas for post-fenugreek treatment, cardiomyocytes were treated with CoCl_2_ and were incubated for 12 hr followed by fenugreek and CoCl_2_ treatment for 24 hr. The fenugreek treatments were carried out in a 96-well culture plate as 15 000 cells/well were seeded and incubated with various concentrations of fenugreek; 10 µg/ml, 20 µg/ml, 40 µg/ml, 80 µg/ml, 160 µg/ml, and 320 µg/ml; at different time-points; 24, 48, and 72 hr. The untreated group (without CoCl_2_ and fenugreek treatment) served as control. The same treatments were used to investigate whether fenugreek inhibits the apoptotic signaling of cardiomyocytes treated with CoCl_2_ and the effect on beating rate and calcium changes of the CoCl_2_-induced hypoxia. 

***Determination of bioactive compounds in fenugreek***

Gas chromatography-mass spectrometry (GCMS) analysis was performed on the fenugreek solution using an Agilent Technologies Instrument System (GCMS7890B). Helium gas was used as the carrier gas at a constant flow rate of 1 ml/min, injector temperature 250 °C; oven temperature was programmed from 110 °C (isothermal for 2 min), with an increase of 10 °C/min to 200 °C, 5 °C/min to 280 °C, ending with a 9 min (17). The total GC running time was 36 min. The chromatogram was obtained through analysis by MassHunter software (Agilent Technologies, USA). 

***Cell viability assay***

The cell viability in each treatment group was measured using CellTiter 96^®^ AQueous One Solution reagent according to the manufacturer’s instructions (Promega^®^). The 96-well assay plates were incubated with CellTiter 96^®^ AQueous One Solution reagent at 37 °C with 95 % O_2_ and 5 % CO_2_ for 1 hr. After 1 hr, the formazan produced by dehydrogenase enzymes was measured by the optical density (OD) values recorded at 490 nm proportionate to the number of viable cardiomyocytes in the culture. The survivability average value (%) was calculated by normalizing a ratio between the absorbance of treated cells and untreated cells, multiplied by 100. The experiment was carried out in triplicates. Results were presented as fenugreek concentration against cell viability in a scattered graph.

***Beating assessment and morphological structure***

A beating assessment was performed according to Clark *et al*., (1991) who visually recorded the beating rate using a bright field microscope (Olympus, USA) (18). Cardiomyocytes were viewed before treatment and after treatment under a light microscope to visually analyze the morphology structure of well-established cardiomyocytes in cell culture. A small network of cardiomyocytes normally composed of 2 to 4 cardiomyocytes was selected for assessment. If there was no contraction observed in the selected field over 30 sec the culture was recorded as negative for beats. To eliminate biased, multiple readings from the same culture were performed. The beating rate of each cardiomyocyte’s network was assessed three times. Each beating rate was counted for 1 min. In addition, in each well for all treatments, three different clusters were selected (technical replicates) and averaged as one reading. The experiment was repeated 5 to 6 times (5-6 biological replicates). Results were presented as beats per min (bpm) against treatment. A paired t-test was used to compare between treatments.

***Calcium signaling assay***

The calcium signaling assay was carried out using Fluo-4 Direct™ Calcium Assay Kit according to the manufacturer’s protocol. An equal volume of 2× Fluo-4 Direct™ calcium reagent was directly added to cells in the assay plates and incubated at 37 °C with 95 % O_2_ and 5 % CO_2_ for 60 min. The fluorescence was measured using VarioskanFlash (Thermo Fisher Scientific) for excitation at 494 nm and emission at 516 nm, respectively. The measurement of intracellular calcium was observed at relative fluorescence unit (RFU; Maximum (max) response – Minimum (min) response was normalized with min response).

***Real-time polymerase chain reaction***

Cardiomyocytes from treated and untreated cell culture plates were detached using trypsin. The RNAs were extracted using TRIzol™ reagent (Thermo Fisher Scientific) according to the manufacturer’s protocol. The colorless RNA was carefully removed and transferred into a new microcentrifuge tube. Nuclease-free water was used to dissolve the RNA pellet on the hotplate (60 °C) for 5 min. The RNA was stored at -80 °C for further analysis. ReverTra Ace^TM^ qPCR RT Master Mix with gDNA remover (FSQ-301, Toyobo, Japan) was used to synthesize cDNA according to the manufacturer’s protocol. THUNDERBIRD™ SYBR^®^ qPCR Mix was used for qPCR. In this study, different batches of cardiomyocytes isolation were used (n = 4), and qPCR was performed on all 9 targeted genes that are responsible for hypoxia (*HIF-1α, GLP-1r, ERK5, and IP3R*) and apoptosis (*Bcl-2, caspase-3, caspase-8, caspase-9, and caspase-12*) with 1 housekeeping gene (*gapdh*) as control. qPCR was carried out in a 12.5 µl reaction. Primers were present at 5 µM, and 0.75 µl of cDNA template was added to the reaction. qPCR was performed in a StepOnePlus^TM^ Real-time PCR System (Applied Biosystems) using programs: Pre-denaturation at 95 °C for 10 min (1 cycle), denaturation at 95 °C for 1 min, annealing at 60 °C for 15 sec and extension at 95 °C for 15 sec (40 cycles) and hold at 4 °C. Data were analyzed using fold change expression against untreated groups. 

***Statistical analysis***

Data were analyzed using the SPSS software package where ANOVA was used for parametric data and Kruskal Wallis for non-parametric data. The statistical significance was set at *P*<0.05 and *P*<0.01, n = 6. Data from more than three (n ≥ 4) isolations of primary cell culture were collected to eliminate the biological error. Data collection of the isolations was combined to create a concrete result to represent the *in vitro *technique analysis. The calculation for mean and standard deviation (SD) was recorded to present the variation of mean differences in data collection. Calculating the confidence interval at 95% difference represents the true value means between the upper and lower limits of the sample sizes. The normality test for data distribution was determined by the Kolmonogrov-Smirnov test. A paired t-test was used to determine the means differences that would affect the variation in data collection for the significant difference between the two conditions. The *P*-value (‘α’ is 0.05) was presented to show the likelihood to produce deviation.

## Results

***Bioactive compounds of food-grade absolute fenugreek natural***

Various compounds were detected in fenugreek obtained from Sigma Aldrich using Gas Chromatography-Mass Spectrometry (GCMS) analysis, which utilized the Mass Spectral Library provided by the National Institute of Standards and Technology (NIST) index library in the United States of America (USA). These compounds are validated for their importance in traditional and modern medicines. The chromatogram obtained from GCMS analysis identified 14 major phytocompounds in fenugreek and their corresponding pharmacological activities (19), which are summarized in Table 2. The identification of these phytochemical compounds was based on various factors such as retention time, peak area, molecular weight, and molecular formula. Fenugreek contained several major phytocompounds such as lactose (36.06%), 1-Heptatriacotanol (2.27%), fenretinide (2.17%), hexadecanoic acid (9.75%), palmidrol (6.83%), linoleic acid ethyl ester (24.45%), ethyl iso-allocholate (3.94%), glycerol 1-palmitate (31.53%), vitamin E (30.78%), and betulin (57.48%). Additionally, fenugreek had high phytosterol content such as cholesterol (10.73% peak area), campesterol (24.95%), stigmastanol (3.92%), and gamma-sitosterol (81.33%).

***Effects of fenugreek on cardiomyocytes cell viability***

Our study shows that the viability of normal cells is not affected by fenugreek. As shown in Table 3, the viability of cardiomyocytes remained at more than 100% even at low or high concentrations (0–320 µg/ml) of fenugreek used. To maintain cardiomyocyte viability throughout the study, we used 24 hr of fenugreek treatment at 160 µg/ml. The viability of cardiomyocytes after 24 hr of incubation with fenugreek (101% ± 0.01) was similar to that of untreated cardiomyocytes, while a significant reduction in viability was observed in cardiomyocytes treated with CoCl_2_.

***Fenugreek effects on beating frequencies, morphological structures, and heart rate markers in hypoxic cardiomyocytes***

**Figure 1:**
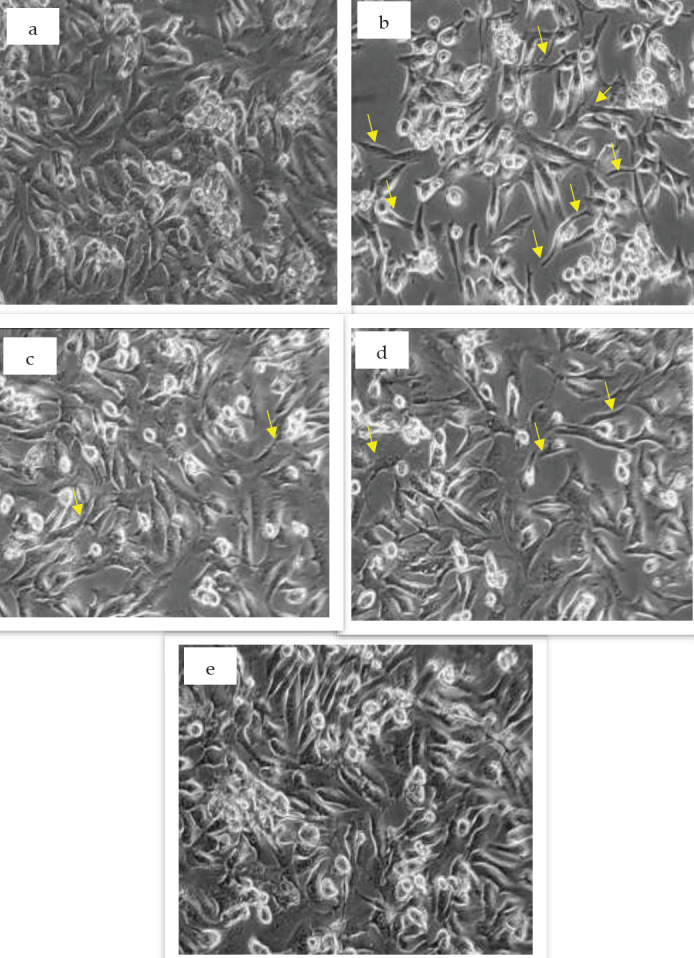
Morphological structures of cardiomyocytes treated with fenugreek (a), CoCl_2 _(b), pre-fenugreek (c), and post-fenugreek (d) against untreated (e)

Compared to the untreated cardiomyocytes (Figure 1e), those treated with fenugreek (Figure 1a) exhibit a more synchronized and well-organized striated structure, whereas those treated with CoCl_2_ (Figure 1b) display a significantly larger gap between small clusters, resulting in reduced synchronization of beating frequencies due to a lower number of present cardiomyocytes. The impact of CoCl_2_ on pre-fenugreek (Figure 1c) and post-fenugreek (Figure 1d) treatment groups differs in terms of the number of cardiomyocytes present, with the former having more than the latter. Moreover, CoCl_2_ treatment (Figure 1b) results in fewer viable cells than untreated cardiomyocytes. 

**Figure 2:**
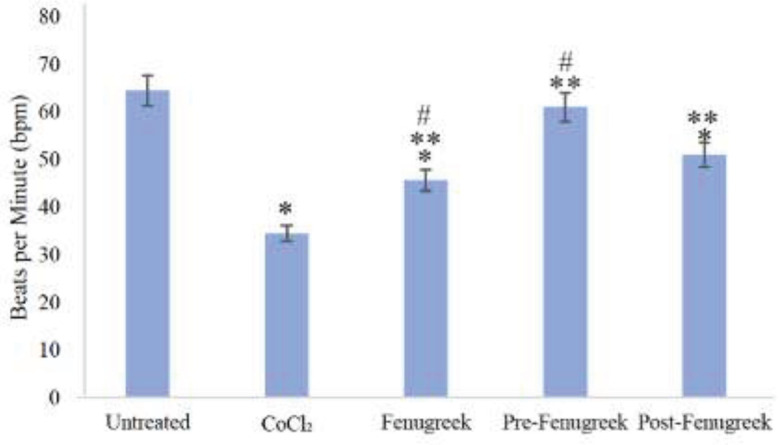
Beating rate (beat per minute; bpm) significantly decreased in CoCl_2_-induced hypoxia (160 µg/ml) in treated cardiomyocytes as compared to untreated

This study revealed that CoCl_2_ treatment significantly reduced the beating rate of cardiomyocytes, with the beating frequencies of the CoCl_2_ treated group (Mean ± SD; 35 bpm ± 0.707; *P*=0.002) decreasing by 53%. On the other hand, fenugreek (46 bpm ± 0.577; *P*=0.01) and post-fenugreek (51 bpm ± 1.414; *P*=0.01) treated groups showed a significant difference compared to the untreated group (65 bpm ± 0.707) (Figure 2). Moreover, beating frequencies of the fenugreek (46 bpm ± 0.577; *P*=0.004), pre-fenugreek (61 bpm ± 1.414; *P*=0.001), and post-fenugreek (51 bpm ± 1.414; *P*=0.001) treated groups were significantly increase compared to the CoCl_2 _treated group (35 bpm ± 0.707). Furthermore, when comparing the pre-fenugreek treated group (61 bpm ± 1.414; *P*=0.001) with the post-fenugreek (51 bpm ± 1.414; *P*=0.03), a significant difference was observed. The results indicate that CoCl_2_ treatment led to a decrease in the beating heart rate of cardiomyocytes, while fenugreek treatment only caused a slight difference in the heartbeat compared to the normal rate observed in untreated cardiomyocytes (Figure 2).

The relative expression levels of the *ERK5* gene were determined by qRT-PCR. In this experiment, the cardiomyocyte GAPDH gene was used as the control. The expression of *ERK5* in the fenugreek treatment group was found to be elevated by 0.23-fold, in comparison to the CoCl_2_, pre-fenugreek, and post-fenugreek treatment groups (Table 4).

The heart rate of cardiomyocytes treated with CoCl_2_ decreased, while those treated with fenugreek showed only a slight deviation from the normal rate observed in untreated cells (Figure 2). Additionally, *GLP-1r* gene expression was low in CoCl_2_, pre-fenugreek, and post-fenugreek-treated cardiomyocytes (Table 4). In contrast, the expression of *GLP-1r* in the fenugreek-only treatment group increased by 0.67-fold compared to the other treatment groups. Up-regulation of *GLP-1r *expression in post-fenugreek-treated cardiomyocytes by 0.05-fold, compared to the pre-fenugreek group, was also observed.

***Fenugreek effects on intracellular calcium ions [Ca*** ^2+^ ***]***_i_ ***in hypoxic cardiomyocytes***

**Figure 3:**
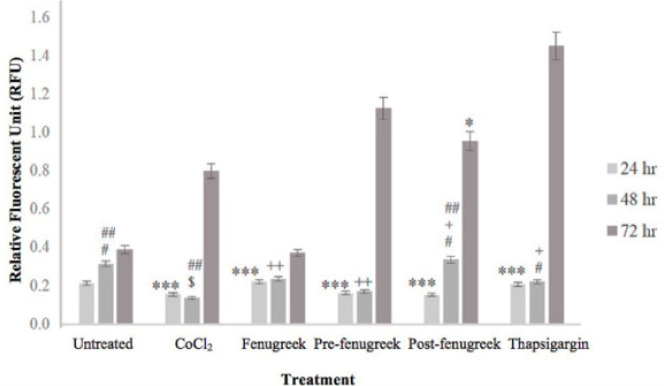
Changes in [Ca^2+^]i values were observed at 24 hr as compared to 48 hr and 72 hr for all treated and untreated cardiomyocyte groups

The inositol 1,4,5-trisphosphate receptor (IP3R) is a type of calcium channel that is ubiquitously expressed in all tissues of the body. In the fenugreek treatment group, the expression of the* IP3R* gene was up-regulated by 0.244-fold compared to other groups, in relation to untreated cardiomyocytes (Table 4). The levels of intracellular calcium ions [Ca^2+^]_i_ were measured at 24 hr in the untreated cardiomyocytes group which was found to be 0.212 relative fluorescence unit (RFU), whereas in treatment groups; CoCl_2_ (0.155 RFU; *P*-value = 0.005), fenugreek (0.220 RFU; *P*-value = 0.005), pre-fenugreek (0.210 RFU; *P*-value = 0.003), post-fenugreek (0.151 RFU; *P*-value = 0.002) and TG (0.206 RFU; *P*-value = 0.005), respectively (as illustrated in Figure 3). After 48 hr, the [Ca^2+^]_i_ levels increased to 0.313 RFU in the untreated cardiomyocytes group, with slight changes observed in all treatment groups: CoCl_2_ (0.136 RFU; *P*-value = 0.07), fenugreek (0.370 RFU; *P*-value = 0.02), pre-fenugreek (0.169 RFU; *P*-value = 0.01), post-fenugreek (0.335 RFU; *P*-value = 0.32) and TG (0.218 RFU; *P*-value = 0.05). However, after 72 hr, higher [Ca^2+^]_i_ levels were observed, with the untreated cardiomyocytes showing 0.387 RFU, and treatment groups CoCl_2_ (0.796 RFU; *P*-value = 0.44), fenugreek (0.370 RFU; *P*-value = 0.12), pre-fenugreek (1.124 RFU; *P*-value = 0.78), post-fenugreek (0.954 RFU; *P*-value = 0.22), and TG (1.449 RFU; *P*-value = 0.84). It was also noted that longer incubation of CoCl_2_ on cardiomyocytes resulted in increased [Ca^2+^]_i_ levels.

***Protective effect of fenugreek on hypoxic cardiomyocytes cell death pathway***

In CoCl_2_-induced hypoxia, the expression of *HIF-1α* was up-regulated by 0.97-fold in comparison to untreated cardiomyocytes, as indicated in Table 4. The CoCl_2_ treatment establishes a hypoxic microenvironment in cells, unlike the fenugreek treatment group. In cells treated with fenugreek, a low expression of *HIF-1α* was observed. This suggests that fenugreek is capable of inhibiting *HIF-1α* expression, which is beneficial for the cells as it helps to minimize the effects of hypoxia injury.

***Fenugreek inhibits the apoptosis gene signaling pathway***

Compared to other groups, the fenugreek treatment group showed a higher expression of the apoptotic gene *Bcl-2*. Conversely, the expression of *caspase-8*, another apoptotic gene, was down-regulated in the fenugreek and pre-fenugreek treatment groups in relation to untreated cardiomyocytes. In contrast, CoCl_2_ and post-fenugreek treatments led to up-regulation of *caspase-8* in comparison to untreated cardiomyocytes. Notably, the post-fenugreek treatment group exhibited a higher expression of *caspase-8* (0.76-fold), indicating that the cells suffered ischemic injury as a result of early CoCl_2_ treatment. The fenugreek and pre-fenugreek treatment groups showed low expression of caspase-8, indicating that the cells were well protected. The expression of *caspase-9* was up-regulated in the pre-fenugreek treatment group (0.51-fold) when compared to the post-fenugreek treatment group. Meanwhile, the fenugreek treatment group exhibited a 9.05-fold up-regulation of *caspase-12* compared to other groups, and up-regulation of *caspase-3* was observed in CoCl_2_-treated cells compared to other treatment groups, as shown in Table 4.

## Discussion

For centuries, medicinal herbs and spices have been used to treat chronic diseases, such as diabetes, cardiovascular diseases, and skin diseases. Traditional medicine is relied upon by about two-thirds of the world’s population for their primary medical needs (20). Fenugreek is one of the most popular medicinal spices, with numerous phytochemical compounds that offer pharmacological benefits against various diseases. Our study identified fourteen major phytocompounds with high phytosterol content. Phytosterols play a crucial role in reducing cholesterol absorption in the body (21) and inhibiting the absorption of cholesterol by forming micelles, thus reducing the risk of cardiovascular disease (22).

Clinical trials have shown that consuming phytosterols can lead to 10–20% reduction in the risk of CVD (23). Fenugreek, in particular, has been shown not to affect the cell viability of normal cells, maintaining cardiomyocyte viability at more than 100% at low or high concentrations. Toxicological data reliability assessments have demonstrated that consuming fenugreek seeds in the form of powder or oil is safe (24). Fenugreek reduces the risk of hardening arteries by effectively lowering plasma glutathione levels and glutathione reductase activity (25). Furthermore, the oral and intraperitoneal administration of fenugreek in male Sprague-Dawley rats has been shown to improve blood glucose, renal, and liver functions, further demonstrating the safety and efficacy of fenugreek (6). 

The cardiomyocytes are responsible for maintaining the heartbeat and rhythm of the heart. To create a hypoxic environment in the cardiomyocytes, we utilized CoCl_2_ in this study. Hypoxia has been found to affect the heart rate variability in healthy human adults (26), leading to abnormal intrinsic cardiac rhythm when cardiorespiratory phase synchronization is disrupted during acute hypoxia (27). The pathologic mechanism of CoCl_2_ involves oxidative stress, inflammation, and apoptosis, which can be reduced by enzymatic and non-enzymatic antioxidants (28). To study contractility, electrophysiology, and morphology, it is crucial to limit the number of viable cardiomyocytes in the experimental model since neonatal rat ventricle cardiomyocytes do not typically proliferate in culture media (29). Mimicking hypoxia with CoCl_2_ in cardiomyocytes resulted in increased actin fiber breakage and decreased fiber density arrangement (30), potentially leading to cell death through autophagy and apoptosis (31). However, intermittent hypoxia was found to increase myocardial autophagy in male mice cardiomyocytes, which is an adaptive measure to prevent endoplasmic reticulum (ER)-stress and cell death from ischemic injury (32).

CoCl_2_ is a chemical that mimics the microenvironment of oxygen depletion inside cells and functions as an inducing agent for oxidative stress (33). Co (II) reacts with hydrogen peroxide in the body and directly produces reactive oxygen species (ROS), which decrease cardiomyocyte viability and alter DNA irreversibly through modulation of calcium influx that affects cell survival (34). Additionally, cardiomyocytes require a large supply of energy from mitochondria during contraction, and interference with mitochondrial function can also disturb their contraction. However, fenugreek was observed to counteract the effect of CoCl_2_. In the fenugreek-treated group, CoCl_2_-induced hypoxia reduced the beating rate by only 47% compared to untreated cardiomyocytes.

Fenugreek is rich in various compounds, such as diosgenin, vitamin E, and steroidal saponin which contribute significantly to cardioprotection (35). For example, diosgenin from *Dioscorea bulbifera*, also known as air potato or aerial yam, has been found to enhance the survival of H9c2 cardiomyoblast cells after hypoxia-reoxygenation injury by decreasing the release of lactase dehydrogenase and modulating pro-survival (B-cell lymphoma 2; Bcl-2) and pro-death (Bcl-2–associated X protein; Bax) molecules, thereby protecting cardiac cells against hypoxia/reoxygenation injury (36). Additionally, the steroidal saponin and diosgenin present in fenugreek have been shown to possess significant antithrombotic properties against atherosclerosis (37). Research has identified diosgenin as a compound that has beneficial effects on various pathophysiological markers, including glucose tolerance, inflammation, insulin actions, liver functions, blood lipids, and cardiovascular health (38).

 The activation of the mitogen-activated protein kinase (MAPK) family signaling pathways, including cellular proliferation, migration, survival, angiogenesis, metabolism regulation, and glucose homeostasis, is induced by ROS. MAPK is a large family that plays a role in the function of adipose tissue (39). Among the subfamily of MAPK, extracellular signal-regulated protein kinase 5 (ERK5) has emerged as an important mediator of heart function (40). In obese or diabetic animal models, the deletion of ERK5 in the heart and the cardiac-specific deletion of ERK5 in mice (*ERK5* is conditionally deleted in Nestin-expressing neural stem cells; *ERK5*-CKO) result in reduction of cardiac contractility and mitochondrial abnormalities followed by suppression of fuel oxidation and increased oxidative damage upon high-fat diet (41). 

Table 4 shows that CoCl_2_-treated cardiomyocytes exhibited mitochondrial dysfunction due to the low expression of *ERK5* compared to fenugreek-treated cardiomyocytes. However, there were only slight differences in *ERK5* expression between pre-fenugreek treatment and post-fenugreek treatment. This suggests that fenugreek treatment after CoCl_2_ treatment protects the cells by up-regulating *ERK5* expression. 

The involvement of *GLP-1r* expression in fenugreek and CoCl_2_-treated cardiomyocytes was further investigated in the study due to its responsibility in controlling heart rate in mice cardiomyocytes by regulating chronotropic activity (42). Results showed that *GLP-1r* expression was up-regulated by 0.67-fold in the fenugreek-only treatment group compared to the other treatment groups. Furthermore, post-fenugreek-treated cardiomyocytes exhibited an increase in positive chronotropic activity compared to pre-fenugreek-treated cells. Positive chronotropic activity indicates an increase in heart rate, while negative chronotropic activity indicates a decrease in heart rate activity. High expression of *GLP-1r* agonist has been reported to reduce cardiovascular events such as heart failure and atherosclerosis in diabetic patients (43).

The detection of calcium ions (Ca^2+^) involves a visible excitation wavelength with high sensitivity and large fluorescence increase resulting from Ca^2+^ binding. Ca^2+^ plays a crucial role in heart contraction (44) by causing Ca^2+^ changes at the cellular level through excitation-coupling of cardiomyocytes. We observed almost similar [Ca^2+^]i values in untreated, fenugreek-treated, and pre-fenugreek-treated cardiomyocytes after 24 hr.

However, at 48 hr and 72 hr, we observed high throughput of Ca^2+^ transient in fenugreek-treated cardiomyocytes. To chemically induce endoplasmic reticulum stress, we used the non-competitive inhibitor of sarco/endoplasmic reticulum Ca^2+^-ATPase (SERCA) and Thapsigargin (TG), as a positive control. High concentrations of TG inhibited cell proliferation. SERCA is important in the dynamic changes of Ca^2+^ levels for muscle relaxation (decrease Ca^2+^ level) and contraction (reload Ca^2+^ into sarcoplasmic reticulum). A high concentration of TG inhibits cell proliferation. 

IP3Rs is known to regulate cardiomyocyte function in response to neurohormonal agonists, which are implicated in cardiac disease. In neonatal ventricular cardiomyocytes, attenuation of IP3R expression inhibits endothelin-1, a potent vasoconstrictor that is crucial for muscle contractility, vascular tone, and cardiomyocyte growth and survival (45). Fenugreek-treated cardiomyocytes show an increase in Ca^2+^ due to up-regulation of IP3R expression at 24 hr, indicating that fenugreek protects cardiomyocytes against the effects of CoCl_2_.

CoCl_2_ is a hypoxia-mimicking agent which induces up-regulation of HIF-1α, establishing a hypoxic microenvironment in treated cells. In contrast, fenugreek treatment resulted in low expression of *HIF-1α*, indicating its potential to inhibit *HIF-1α* expression and protect cells against hypoxia injury. In myocardial infarction mice, *HIF-1α* up-regulation specifically in cardiomyocytes results in reduced contractility (46). However, in ischemic heart disease and pressure-overload heart failure, *HIF-1α* activation plays a critical protective role by activating homeostatic mechanisms (47). Interrupted mitochondrial energy production results in irregular oxygen flow in the heart, which is important for cardiomyocyte contraction (48). 

The process of apoptosis involves two main pathways, the intrinsic and extrinsic pathways. The intrinsic pathway is initiated when a cell experiences injury, such as hypoxia, which triggers the HIF-1α protein of the apoptotic pathway. This pathway involves specific proteins such as Bcl-2, Bax, cytochrome c, apoptotic protease activating factor-1 (APAF-1), caspase-9 (initiator caspase), and caspase-3 (executioner caspase). In contrast, the extrinsic pathway is activated outside the cell when conditions in the extracellular environment determine that a cell must die. This pathway involves the expression of specific proteins such as caspase-8 (initiator caspase) and caspase-12 (inflammation caspase) and modulates caspase-3 activation. Ligands are required to activate the death receptor, which then activates caspase-8 and pro-inflammatory signaling (49).

The Bcl-2 protein family includes both pro-apoptotic and anti-apoptotic proteins that determine whether mitochondria initiate cell death. Anti-apoptotic Bcl-2 regulation has been shown to protect against various cardiac pathologies (50). In our study, we found higher expression of *Bcl-2* in the fenugreek treatment group compared to the other groups. Up-regulation of *Bcl-2* inhibits the release of cytochrome c and activation of caspase-3/9 in hypoxia/reoxygenation of adult cardiomyocytes, thereby protecting the cells against the mitochondrial-apoptosis pathway (51).

The extrinsic pathway of apoptosis requires initiator caspases or recruitment caspases. Caspase-8 is an initiator caspase activated by the ligand death receptor of the extrinsic pathway, which can eventually lead to inflammation (52). In the fenugreek and pre-fenugreek treatment groups, low expression of *caspase-8* was observed, indicating that the cells were well protected. However, an increase in *caspase-8* expression without any association with caspase-3 involves the Fas-associated protein with death domain (FADD) and is associated with an increased incidence of ischemic stroke (53).

The pre-fenugreek treatment group exhibited up-regulation of *caspase-9* compared to the post-fenugreek treatment group, suggesting that the concentration of fenugreek used may not have been sufficient for the treatment. Caspase-9 activation occurs through the dimerization of pro-caspase monomers, forming an apoptosome, and does not require an activation inducer (54). Apoptosome is composed of proteins that are triggered by the release of cytochrome c from mitochondria due to stress build-up in cells. Caspase-9 is involved in cardiac fibrosis and dysfunction through the generation of oxidative stress via mitochondria-induced apoptosis (55). 

Cell death caused by hypoxia in the myocardium plays a vital role in maintaining cellular proliferation and homeostasis as an adaptive response (56). Hypoxia leads to protein misfolding which disrupts the disulfide bonds in the endoplasmic reticulum (ER), causing stress that activates the unfolded protein response (UPR). During hypoxia, UPR activates caspase-12, which is involved in the pro-apoptotic response. The expression of the *caspase-12* gene was found to be up-regulated in cardiomyocytes treated with fenugreek before and after hypoxia. Caspase-12 plays a crucial role in apoptosis by disrupting the ER’s calcium balance and causing an imbalance in demand and capacity during hypoxia (57). The expression of the *caspase-12* gene is categorized as an initiator caspase, which triggers an inflammatory response due to the accumulation of unfolded proteins in the ER and may not necessarily be involved in executioner cell death (58).

The activation of initiator caspases, such as caspase-8 and caspase-9, is necessary for the activation of the well-known apoptotic protein, caspase-3. Caspase-3 is responsible for regulating DNA fragmentation and cytoskeletal protein degradation during apoptosis. Our findings indicate that CoCl_2_ treatment leads to higher expression of caspase-3 than the other treatment groups. Activation of caspase-3 has been associated with ischemic cardiomyopathy and idiopathic dilated cardiomyopathy in heart failure patients (59). Under hypoxic conditions, H9c2 cardiomyocytes have been shown to induce apoptosis through up-regulation of cleaved caspase-3, which can be inhibited by compounds containing salvianolic acid B, known for their antioxidant properties, such as those found in *Radix Salvia miltiorrhiza*, red sage, and Danshen roots (60). Our findings indicate that fenugreek can inhibit apoptosis by virtue of its antioxidant properties, which help to counteract the effects of hypoxia-induced apoptosis. 

**Table 1:** Digestion and incubation steps in isolation of neonatal sprague dawley rat cardiomyocytes

**Digestion/Collection**	**1**	**2**	**3**	**4**	**5**
Enzyme solution (ml)	10	8	8	6	6
Incubation time (min)	20	25	25	15	20
Shaking incubator speed (rpm)	200	180	180	200	280

**Table 2:** Phytochemical compounds in fenugreek and its pharmacological activities

**No.**	**Name of the compound**	**RT (min)**		**Molecular weight (g/mol)**		**Molecular formula**	**Score**	**Peak area %**	**Height, mAU**	**Pharmacological activities **
**1**	Lactose	7.912 / 10.870	342.30		C_12_H_22_O_11_		77.01	36.06	5489951	Source of energy; reduce heart disease risk
**2**	1-Heptatriacotanol	11.378	537.00		C_37_H_76_O		75.84	2.27	656591.2	Antimicrobial; anti-hypercholesterolemic effects
**3**	Fenretinide	12.292	391.55		C_26_H_33_NO_2_		76.6	2.17	397947.4	Anticancer and chemoprotective drugs
**4**	Hexadecanoic acid	14.611	256.43		C_18_H_36_O_2_		86.74	9.75	6026456	Antioxidant; antimicrobial; cardioprotective
**5**	Palmidrol	16.546	299.50		C_18_H_37_NO_2_		78.19	6.83	7175558	Analgesia, neuroprotection; anti-epileptic; anti-cancer; cardioprotective
**6**	Linoleic acid ethyl ester	16.941	308.50		C_20_H_36_O_2_		84.53	24.45	7984330	Anti-inflammatory; lowered heart rate
**7**	Ethyl iso-allocholate	21.076	436.63		C_26_H_44_O_5_		59.27	3.94	1485661	Antimicrobial
**8**	Glycerol 1-palmitate	21.808	330.50		C_19_H_38_O_4_		76.73	31.53	9229550	Cardiac energy production
**9**	Cholesterol	30.291	386.65		C_27_H_46_O		84.58	10.73	7011332	Prevent and reverse heart diseases
**10**	Vitamin E	30.613	430.71		C_29_H_50_O_2_		85.23	30.78	13525155	Antioxidant; cardioprotective
**11**	Campesterol	32.237	400.70		C_28_H_48_O		81.01	24.95	4759987	Inhibits absorption of cholesterol
**12**	gamma-Sitosterol	34.245	414.70		C_29_H_50_O		87.59	81.33	12266783	Antidiabetic
**13**	Stigmastanol	34.520	416.72		C_29_H_52_O		69.18	3.92	900209.3	Anti-cholesteremic
**14**	Betulin	36.092	442.72		C_30_H_50_O_2_		79.77	57.48	7764840	Cardioprotective; anti-inflammatory

The elongated actin of CoCl2-treated cardiomyocytes (yellow arrow) was observed under a light microscope at 200× magnification as a network of cardiomyocytes; representatives of 3 independent observations (n = 3).

**Table 3:** Effect of fenugreek and CoC_l2_ treatment at different concentrations and incubation times on cardiomyocytes’ cell viability

Treatment concentration (µg/ml)	Cell viability (%) at different treatment incubation times					
	24 hr		48 hr		72 hr	
	Fenugreek	CoCl_2_	Fenugreek	CoCl_2_	Fenugreek	CoCl_2_
0	100	100	100	100	100	100
10	100	98	101	97	102	107
20	104	98	101	99	106	109*
40	108*	100	105*	103	96	103*
80	112*	90	104*	93	94	98
160	101	66*	105*	80*	90	86*
320	101	58*	111*	76*	82	74*

* indicates the significance at *P*<0.05 compared to the untreated group

* indicates significance at *P*<0.01 compared to untreated, ** indicates significance at *P*<0.01 compared to CoCl_2_, # indicates significance at *P*<0.05 compared to post-fenugreek; n = 3 independent experiments.

* indicates significance at P<0.05 compared to untreated, ** indicates significance at *P*<0.05 compared to CoCl_2_, *** indicates significance at *P*<0.05 compared to fenugreek, $ indicates significance at *P*<0.05 compared to pre-fenugreek, $$ indicates significance at *P*<0.05 compared to post-fenugreek; n = 3 independent experiments.

**Table 4:** Expression of 9 targeted genes that are responsible for hypoxia (*HIF-1α*, *GLP-1r,* *ERK5*, and *IP3R*) and apoptosis (*Bcl-2*, *caspase-3*, *caspase-8*, *caspase-9 *and *caspase-12*). The ΔΔCt value was determined by subtracting the ΔCt value of the untreated sample from the ΔCt value of the treated sample. Assuming the efficiency is 100%, the fold change due to treatment was described as 2^−ΔΔCt^

**Treatment**	**ΔΔCt, fold change due to treatment**								
	***ERK5***	***GLP-1r***	***IP3R***	***HIF-1α***	***Bcl-2***	***Caspase-3***	***Caspase-8***	***Caspase-9***	***Caspase-12***
**Fenugreek**	2.12, 0.23	0.58, 0.67	2.03, 0.244	1.34, 0.39	1.47, 0.36	1.51, 0.35	1.15, 0.45	0.93, 0.52	-3.18, 9.05
**CoCl**_2_	6.05, 0.02	1.50, 0.35	3.54, 0.086	0.04, 0.97	2.45, 0.18	1.04, 0.49	0.82, 0.57	0.79, 0.58	-2.18, 4.53
**Pre-Fenugreek**	6.58, 0.01	1.85, 0.28	8.66, 0.002	3.48, 0.09	2.59, 0.17	1.76, 0.30	3.17, 0.11	0.96, 0.51	0.73, 0.60
**Post-Fenugreek**	4.28, 0.05	1.60, 0.33	10.14, 0.001	1.99, 0.25	4.55, 0.04	2.09, 0.23	0.39, 0.76	3.32, 0.10	-2.14, 4.41

## Conclusion

Our study has found that treating cardiomyocytes with CoCl_2_ at a concentration of 160 μg/ml for 24 hr creates a hypoxic environment that decreases cell viability and beating rate while up-regulating *HIF-1α* expression. We suggest that this concentration and duration of treatment are optimal for studying the effects of hypoxia on cardiomyocytes. However, fenugreek was found to maintain cell viability and beating even at higher concentrations and can protect against CoCl_2_-induced hypoxia when given before or after treatment. Our study also reveals that fenugreek inhibits apoptosis through up-regulation of *Bcl-2* and down-regulation of executioner *caspase-3* activation. Despite an increase in initiators *caspase-8* and *caspase-9*, a decrease in *caspase-3* gene expression was also observed. Furthermore, our results indicate that fenugreek induces intracellular calcium changes, which may contribute to its cardioprotective effects. Overall, our findings suggest that fenugreek has the potential to be a therapeutic cardioprotective agent and could be used as a daily supplement to prevent ischemic heart injury. Future studies should investigate the changes in protein levels to confirm the findings of this study.

## Authors’ Contributions

PV R and K AKP provided conception and methodology; NI H performed experimental works; PV R, K AKP, M M, and KNS S supervised; PV R, GS H, NH S, and K AKP acquired funding; NI H wrote the original draft; NI H, K AKP, and PV R contributed to writing, reviewing, and editing. 

## Conflicts of Interest

The authors declare no conflicts of interest. The funders had no role in the design of the study, in the collection, analysis, or interpretation of data, in the writing of the manuscript, or in the decision to publish the results.
